# Multi-Year Leaf-Level Response to Sub-Ambient and Elevated Experimental CO_2_ in *Betula nana*

**DOI:** 10.1371/journal.pone.0157400

**Published:** 2016-06-10

**Authors:** Alexandra J. C. Hincke, Tom Broere, Wolfram M. Kürschner, Timme H. Donders, Friederike Wagner-Cremer

**Affiliations:** 1 Palaeoecology, Department of Physical Geography, Faculty of Geosciences, Utrecht University, Utrecht, The Netherlands; 2 Department of Geosciences, University of Oslo, Oslo, Norway; Universidade Federal de Viçosa, BRAZIL

## Abstract

The strong link between stomatal frequency and CO_2_ in woody plants is key for understanding past CO_2_ dynamics, predicting future change, and evaluating the significant role of vegetation in the hydrological cycle. Experimental validation is required to evaluate the long-term adaptive leaf response of C3 plants to CO_2_ conditions; however, studies to date have only focused on short-term single-season experiments and may not capture (1) the full ontogeny of leaves to experimental CO_2_ exposure or (2) the true adjustment of structural stomatal properties to CO_2_, which we postulate is likely to occur over several growing seasons. We conducted controlled growth chamber experiments at 150 ppmv, 450 ppmv and 800 ppmv CO_2_ with woody C3 shrub *Betula nana* (dwarf birch) over two successive annual growing seasons and evaluated the structural stomatal response to atmospheric CO_2_ conditions. We find that while some adjustment of leaf morphological and stomatal parameters occurred in the first growing season where plants are exposed to experimental CO_2_ conditions, amplified adjustment of non-plastic stomatal properties such as stomatal conductance occurred in the second year of experimental CO_2_ exposure. We postulate that the species response limit to CO_2_ of *B*. *nana* may occur around 400–450 ppmv. Our findings strongly support the necessity for multi-annual experiments in C3 perennials in order to evaluate the effects of environmental conditions and provide a likely explanation of the contradictory results between historical and palaeobotanical records and experimental data.

## Introduction

Stomatal frequency analysis is a well-established proxy technique used to reconstruct atmospheric CO_2_ dynamics from leaf cuticle morphology of fossils and herbarium leaf specimens [1,2], encompassing the broad variation in the estimated Cenozoic CO_2_ range from glacial minima of 180 ppmv [3] to maxima of over 1000 ppmv during past ‘hothouse’ conditions [4,5]. Based on the strong link between leaf cuticle morphology and CO_2_, the coupling of stomatal conductance and CO_2_ has been investigated in an effort to further develop the predictive capacity of CO_2_ imprints in fossil leaf remains [6–8]. The 120 ppmv industrial CO_2_ increase is commonly used to calibrate the acclimation of leaf cuticle morphology through the study of historical leaf collections from herbaria [9–11] and leaf fragments preserved in shallow peat sequences [2,9]. In the resulting high-resolution time-series data the majority of woody C3 species studied show a quantifiable reduction of stomatal frequency, changes in stomatal geometry, and down-regulation of structural stomatal conductance [12–14] with increasing atmospheric CO_2_. Stomatal analysis is particularly valuable for our understanding of CO_2_ dynamics in periods preceding ice core-based records, the consequences of on-going future CO_2_ increase [15,16], and to evaluate the active role of vegetation in the hydrological cycle under past and future CO_2_ dynamics [13].

For additional information and calibration of leaf-level response beyond the historical 280–400 ppmv CO_2_ range, controlled-environment CO_2_ experiments are required. While historical datasets show a clear reduction in stomatal parameters over the pre-industrial to present CO_2_ range, morphological adjustment data from woody C3 plants from experimental free-field and growth chambers for past low and projected future high CO_2_ concentrations are largely ambiguous [17,18]. Of the experiments with set-ups constraining the stomatal response under sub-ambient CO_2_, these generally do not show consistent responses in terms of stomatal frequency adjustments to CO_2_ [17,19].

Experimental validation of structural changes in stomatal conductance, as observed in time-series studies, has become a research focus only recently. The few studies on structural stomatal conductance changes available for the pre-industrial to elevated CO_2_ range reveal significant down-regulation from sub-ambient to ambient CO_2_ and levelling off between ambient to elevated CO_2_ [20] (Hincke et al., *submitted*). Although the non-linearity of stomatal frequency and associated stomatal conductance response is to date a well-recognised phenomenon [13,16], no comprehensive experimental validation over the complete range of glacial lows to past and potential future high CO_2_ concentrations is available. Despite the inherent uncertainties in stomatal frequency and structural stomatal conductance records based on fossil or historical leaf samples, all show comparable trends in response to CO_2_, however, testing and validation of leaf morphological acclimation in C3 woody species in experimental set-ups is difficult. It may be the case that certain aspects of the complementary experimental set-ups themselves do not fully capture the long-term adaptive response of stomatal parameters, particularly in perennial C3 plants, to the full range of CO_2_ variability.

We identify and investigate two major issues in short-term controlled-CO_2_ experiments. Firstly, in short-term growth experiments covering less than one full growing season, leaf matureness may be incomplete at the time of sampling. In *Osmunda regalis* leaves stomatal parameters increased during the first 10–20% of leaf development, stabilizing at 30% of total leaf size [21]. In *Eucalyptus regnans* stomatal frequency decreased from 56 until 113 days after emergence, with stomatal initiation continuing until 70% of full leaf expansion [22]. Epidermal cells continue to expand with leaf size; thus, leaves which are not mature may not represent the full expression of stomatal parameters required to calibrate a CO_2_ response. Sampling of leaves during ontogeny, before matureness, potentially does not reflect their full expression and may skew analysis of the stomatal response to CO_2_.

Secondly, we posit that experiments which cover a single growing season only reflect a limited response to pre-set conditions which are extremely different from ambient CO_2_ levels. Monitoring studies on annually-collected leaves of mature birch trees show that leaf cuticle morphology adjustments takes place in multiple successive leaf generations in response to annual CO_2_ increments of approximately 2 ppmv CO_2_ per year [11,23]. A significant response from mature leaves to the next generation has also been experimentally demonstrated for *Arabidopsis* using CO_2_-controlled cuvettes to expose individual leaf generations to high CO_2_ [24]. While the capacity for generational CO_2_ signalling is evident from natural and experimentally-grown leaf material [25], there is still no proof that leaf adjustment captures the full change of morphological response to CO_2_ within one leaf generation. We hypothesize that the apparently ambiguous response in previous free-field and growth chamber studies can partially be explained by the short-term nature of those studies. If the acclimation of leaf-level parameters to experimentally-adjusted CO_2_ conditions occurs over more than one growth season, and thus over multiple leaf generations, a clearer response is likely to be evident in experiments carried out over multiple growth season with the same individual plants.

In order to improve the interpretation of leaf morphological CO_2_ signals and validate the stomatal response of woody C3 plants to CO_2_, we carried out growth experiments to test the within- and between-leaf generation response of stomatal characteristics in *Betula nana* (dwarf Birch) over a broad range of CO_2_ concentrations. The experimental CO_2_ levels of 150 ppmv, 450 ppmv, and 800 ppmv CO_2_ encompass a range of full glacial low to predicted future high CO_2_, which is required to provide data supporting palaeoatmospheric CO_2_ reconstructions, and, importantly, also produces data relevant for modelling efforts of structural stomatal conductance-induced hydrological changes through time. We tested the responsiveness of leaf generations initiated externally and *in situ* in order to evaluate potential uncertainties arising from short-term CO_2_ exposure experiments with perennial plants. The repetition of the experiments in a second consecutive year allows us to evaluate the degree to which morphological CO_2_ acclimation is captured in traditional single-season experiments.

## Methods

The Utrecht University Fytotron (REFTECH B.V., Sassenheim) was set up to artificially simulate environmental conditions and monitor plant growth and development. The three available growth chambers are identical and conditions within each chamber are independently controlled. Chambers measured ~3 x ~1.5 m and have a ~2.2 m high ceiling. Each chamber was equipped with four tables measuring 0.75 x 1.5 m and of adjustable height as a surface on which to place plants. CO_2_ within the chambers was controlled by a molecular sieve and pressure swing adsorption (PSA) technology (PG 1500L, CMC Instruments GmbH, Eschborn), removing CO_2_ and H_2_O vapour after the air was filtered to remove water, oil, dust, and aerosols. In the low CO_2_ chamber, CO_2_-free air was mixed into the volume of the room air to achieve the desired low CO_2_ concentration. While working in the chamber, a gas mask attached to a sealed plastic bag to trap exhaled air was used to limit CO_2_ rise in the chamber. The ambient CO_2_ chamber was maintained with an input of outside air. In the high CO_2_ chamber, extra CO_2_ was added from a tank to achieve the desired CO_2_ concentration while maintaining ambient air pressure. The CO_2_ level in the growth chambers was monitored digitally (BMP343 Vaisala GmbH, Bonn). Relative air humidity (rH) was 70%. Light intensity was ~350 μ mol m^-2^ s^-1^ with a 10 hour photoperiod, comparable to a March day in the Netherlands [19]. Temperature was set to vary between day/night 21°C/18°C, which was the lowest possible setting achievable in this set-up.

The set points of environmental conditions inside the Fytotron were agreed amongst the researchers performing experiments in the facility, and limited by the capacity of the chambers. CO_2_ was set at 150, 400, and 750 ppmv. Some variability was inherent in these set points due to design of the chambers, and the actual CO_2_ levels in the chambers fluctuated around means of 160, 450, and 800 ppmv (±50 ppmv in the ambient and high CO_2_ chamber). The low CO_2_ (150 ppmv) setting was selected to replicate absolute minimum CO_2_ levels during the Last Glacial, where CO_2_ may have been as low as 160 ppmv [3]. The 400 ppmv setting attempted to replicate ambient atmospheric CO_2_ level. The 400 ppmv atmospheric CO_2_ mark was reached at Mauna Loa between April and May 2014 [26]. This mark is thus a realistic comparison for current and near-future atmospheric CO_2_ levels. The 750 ppmv setting was selected to represent potential future CO_2_ levels. IPCC scenarios suggest that the 750 ppmv atmospheric CO_2_ mark may be reached as early as 2075. However, as the actual CO_2_ levels in the chamber were closer to 450 and 800 ppmv these values are used throughout the paper.

*B*. *nana* specimens were obtained from a gardening centre (Plantentuin Esveld, Boskoop) as cuttings in individual 1.5 L pots. They had been exposed to global ambient CO_2_ conditions for multiple growing years. They were placed in outdoor greenhouses at the Utrecht University Botanische Tuinen (Botanical Gardens) before being moved first to the ~20°C greenhouse to acclimatise to the temperature, and then to the CO_2_ chambers and control treatment (temperature-controlled greenhouse) prior to budburst. Of a total 50 plants, 13 were placed in each growth chamber and 11 plants in the greenhouse control. Following the growth season in the chambers, when leaves reached senescence and began to drop from the plant, plants were moved first to the ~20°C greenhouse to acclimate to greenhouse conditions for 2–6 weeks and then to an outdoor greenhouse where they were exposed to winter temperatures. This strategy was developed as it was not possible to adjust daylight length or temperature settings to mimic actual long-term seasonal changes in the Fytotron growth chambers. Plants were re-potted in enriched potting soil (Botanische Tuinen, Utrecht) following the 2013 growth season.

Leaves were sampled weekly throughout the growing season. In 2013, five apparently mature leaves were sampled randomly from the plant population in each growth chamber and in the greenhouse. In 2014, 3–5 leaves per plant were sampled. In this study, 141 leaves of *B*. *nana* were analysed for leaf area (LA) and cuticle micro-morphological properties. Sampling dates are referred to as days in the experimental chamber, where 0 is the first day the plants were exposed to experimental CO_2_ conditions (150, 450, and 800 ppmv), on 18/03/2013 and 20/02/2014. Leaves which had reached apparent maturity were sampled rather than newly-developed or developing leaves. Leaves which grew on shoots newly-formed in the growth chambers were preferentially sampled. The leaves were removed from the plant upon sampling and placed into paper envelopes to prevent mould from forming before they could be dried.

LA was measured using a LI-COR LI-3100C Area Meter. Leaves were dried at 70°C for 24–72 hours and dry weight measured with a Sartorius ENTRIS 3202-1S Precision Scale. Approximately 0.5 cm^2^ of material was removed from the central part of each leaf, avoiding the main hydraulic vein, and soaked in a 4% NaHClO_2_ solution for 24 hours. The abaxial (stomatal-bearing) cuticle could then be loosened and removed from the mesophyll, stained with saffranine, and mounted with glycerine jelly on microscope slides. Cuticle measurements and stomatal counting was performed on an Olympus BH-2 optical microscope with the AnalySIS Auto image analysis system (Soft Imaging System GmbH, Germany, v. 5.1) at 660x magnification with a digital image size of 0.0575 mm^2^. Images had a resolution of 2080 x 1544 pixels. Stomatal density (D_S_) and epidermal cell density (D_E_) were measured on seven stomatal-bearing alveoles. Pore length (L_P_), stomatal length (L_S_), guard cell width (L_GC_), cell circumference, and epidermal cell area (CA_E_) were measured on n = 30 cells or stomata for each slide.

Stomatal index (SI) was calculated to correct for the influence of epidermal cell expansion on stomatal frequency [27] (Eq 1). Maximum pore area (a_max_) was calculated in μm^2^ (Eq 2) and maximum stomatal conductance to water vapour (g_smax_) was calculated using a two-end auto-correction for shell diffusion [28] where D_H2O_ is the diffusivity of water vapour (m^2^ s^-1^) and V is the molar volume of air (mol m^-2^ s^-1^) calculated as constants based on the ambient temperature in °C (Eq 3).

$$SI\;\%=100\;\times \;\frac{D_{S}}{D_{S}+D_{E}}$$ (1)

$$a_{max}=\;\frac{\pi \;\times \;L_{GC}}{8}$$ (2)

$$g_{smax}=\frac{\frac{D_{H_{2}O}}{V}\times \;D_{S}\;\times \;a_{max}}{L_{P}+\frac{\pi }{2}\;\times \;\sqrt{\frac{a_{max}}{\pi }}}$$ (3)

## Results

### Leaf ontogeny of *B*. *nana* in G1-1 and G1-2

Leaves from weekly sampling were analysed and mean values of leaf morphological parameters (Table 1) were produced for each sampling date. The data from the first experimental year (G1) clustered in two phases: a primary leaf flush, G1-1, sampled up to and including 66 experimental days and a secondary leaf flush, G1-2, sampled from 73 experimental days onwards (Table 2). The abbreviations for successive leaf generations are summarised in Table 3. Changes for all ontogenetically relevant parameters, with significantly lower LA and significantly higher D_E_ and D_S_ values at 73 experimental days compared to 66 days, were observed in all CO_2_ treatments (Table 2). L_P_ is generally smaller at 73 days than at 66 days. CA_E_ was smaller in the samples from 73 experimental days with the exception of the 450 ppmv treatment where CA_E_ is approximately the same. SI and g_smax_ did not show comparative variability between the two sampling dates.

**Table 1:** Leaf morphological and stomatal parameters. The leaf morphological and stomatal parameters discussed in this paper and their conventional abbreviations and units.

Parameter	Abbreviation	Unit
Leaf area	LA	cm^2^
Stomatal density	D_S_	n mm^-2^
Epidermal cell density	D_E_	n mm^-2^
Epidermal cell area	CA_E_	μm^2^
Stomata index	SI	%
Maximum stomatal conductance	g_smax_	mol m^-2^ s^-1^
Pore length	L_P_	μm
Stomatal length	L_S_	μm
Guard cell width	L_GC_	μm
Maximum pore area	a_max_	m^2^

**Table 2:** Mean leaf parameters at experimental CO_2_ levels in G1-1 and G1-2. Mean leaf parameters at 66 and 73 experimental days. The leaf response clustered into two distinct stages (G1-1 and G1-2) in the first growing season (G1). Error in standard error of the mean. Tests of significance were two-tailed Student’s T-tests assuming unequal variance.

Parameter (unit)	CO_2_ (ppmv)	G1-1 (66 days)	Change	G1-2 (73 days)	P-value
**LA (cm**^**2**^**)**	150	0.88 ± 0.13		0.59 ± 0.07	<0.002
	450	0.94 ± 0.12	>	0.66 ± 0.06	<0.002
	800	1.37 ± 0.15		0.65 ± 0.09	<0.002
**CA (μm**^**2**^**)**	150	1115.41 ± 172.17		962.21 ± 77.08	ns
	450	945.15 ± 60.03	>	998.02 ± 33.42	ns
	800	1212.69 56.91		883.26 ± 61.3	<0.02
**DE (n mm**^**-2**^**)**	150	968.12 ± 70.06		1588.69 ±84.18	<0.001
	450	881.99 ± 76.30	<	1497.45 ± 135.64	<0.001
	800	1013.66 ±61.72		1286.29 ± 86.96	<0.001
**DS (n mm**^**-2**^**)**	150	127.54 ± 12.53		162.82 ± 9.24	<0.002
	450	106.83 ± 9.6	<	163.42 ± 21.46	<0.002
	800	119.88 4.8		147.74 ± 11.35	<0.002
**LP (μm)**	150	20.86 ± 1.31		18.46 ± 0.5	ns
	450	26.82 ± 0.96	>	17.82 ± 0.66	<0.003
	800	21.71 ±0.56		17.41 ± 0.52	<0.003
**gsmax (mol m**^**-2**^ **s**^**-1**^**)**	150	1.05 ± 0.06		1.09 ± 0.06	ns
	450	1.17 ± 0.05		1.08 ± 0.09	ns
	800	1.1 ± 0.05		0.92 ± 0.06	ns
**SI (%)**	150	11.56 ± 0.049		9.31 ± 0.16	<0.04
	450	10.97 ± 0.52	>	9.8 ± 0.49	ns
	800	10.74 ± 0.57		10.32 ± 0.24	ns

**Table 3:** Growth seasons and leaf generations. The distinction of growth seasons and leaf generations as discussed in this paper.

Growing season	Leaf flush	Leaf flush (days)	Data discussed (days)	Leaf generation
2013	First	0–66	66	G1-1
	Second	73–94	80–94	G1-2
2014	No distinction	No distinction	44	G2

**Fig 1:**
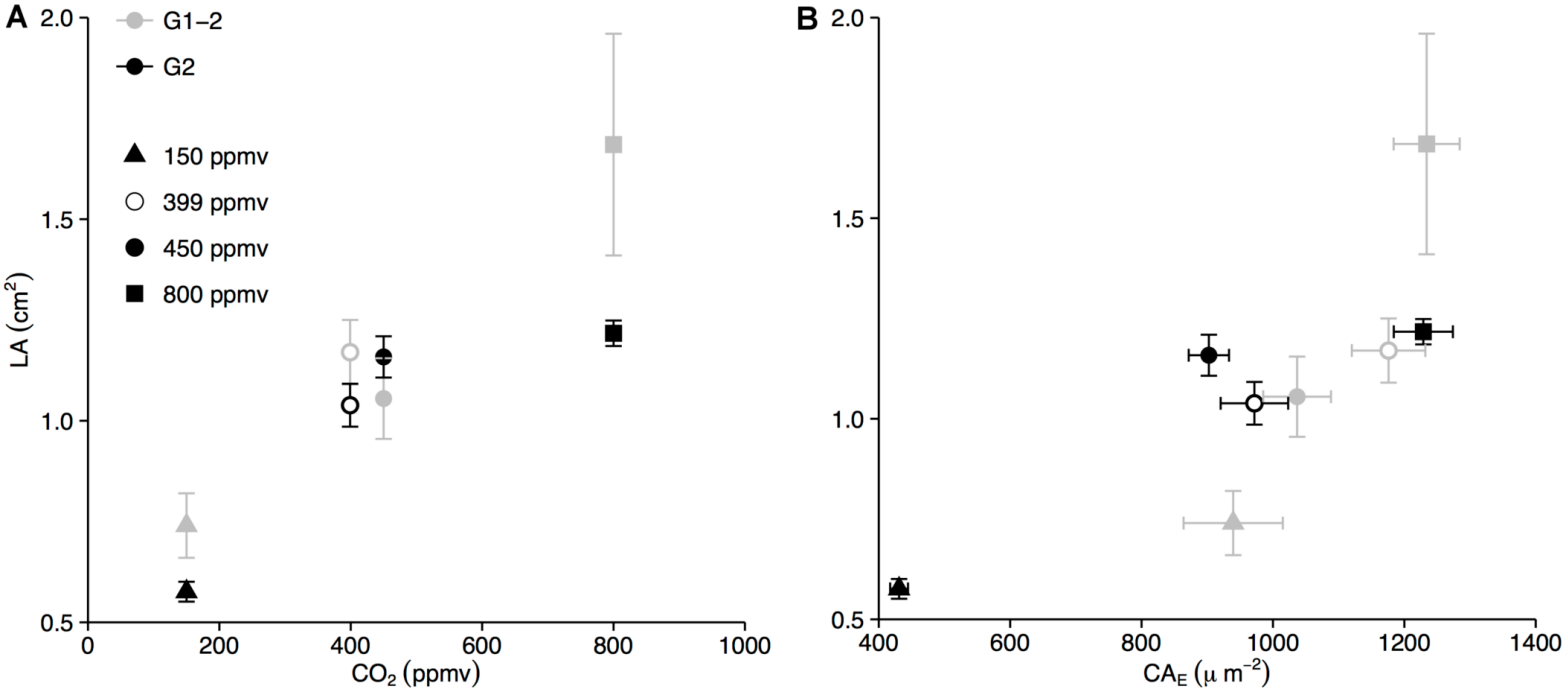
LA of *B*. *nana* leaves increases with CO_2_ and CA_E_ in G1-2 and G2. (A) LA was higher in successive CO_2_ steps in G1-2 and G2. Greenhouse leaves had slightly, but not significantly, larger LA than leaves grown in the 450 ppmv CO_2_ chamber. (B) Larger leaves have larger CA_E_. Error bars represent standard error of the mean.

The secondary leaf flush G1-2 is comprised of leaves which developed entirely *in situ*, that is, inside the growth chamber while exposed only to experimental CO_2_ concentrations. Data presented for G1-2 is the mean of leaves sampled on experimental days 80 and 94 when the leaves produced in G1-2 had reached full maturity and leaf morphological and stomatal parameters were fully expressed. In G1-2, LA increased significantly under increasing experimental CO_2_ concentrations with mean LA of 0.74 cm^2^, 1.06 cm^2^, and 1.69 cm^2^ at 150, 450 and 800 ppmv, respectively. The leaves sampled from the control treatment were slightly larger, with mean LA of 1.17 cm^2^, than leaves from the plants in the ambient experimental CO_2_ growth chamber (Fig 1A).

**Fig 2:**
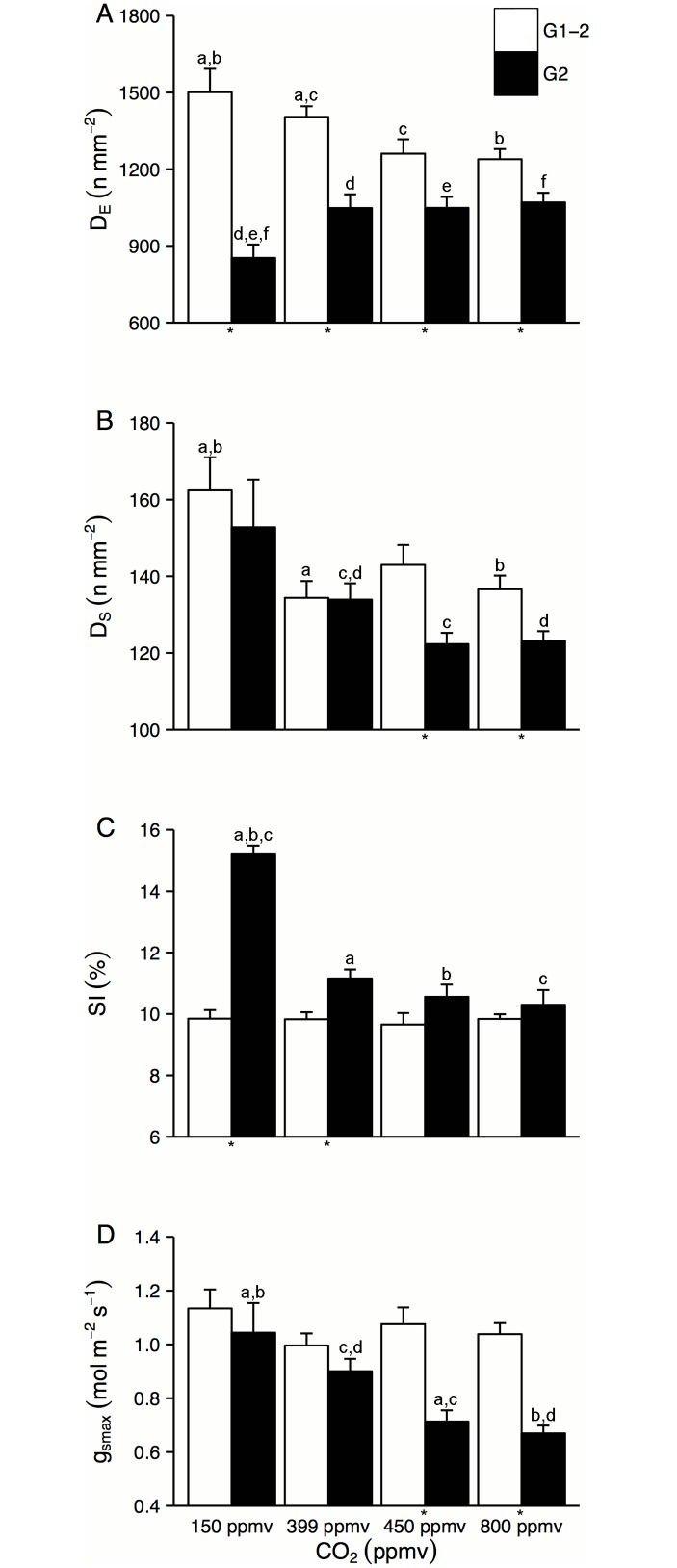
Response of structural stomatal parameters to CO_2_ in G1-2 and G2. (A) D_E_ decreased with CO_2_ in G1-2. In G2, D_E_ is lower at 150 ppmv than in other CO_2_ treatments. (B) D_S_ responded slightly to CO_2_ in G1-2 and shows a levelling-off of the response between 399–450 ppmv in G2. (C) SI did not respond to CO_2_ in G1-2. In G2, the SI response at 150 ppmv was significant with a levelling-off of response around 399 ppmv. (D) The g_smax_ did not respond to CO_2_ in G1-2. In G2, g_smax_ is highest at 150 ppmv, then decreased stepwise with CO_2_ until 450 ppmv, where the response levelled off. Error bars represent standard error of the mean. Asterisks below the bars indicate a significant difference (P<0.05) between parameter data in G1-2 and G2 for that CO_2_ level. Letter codes above the bars indicate significant differences; bars which share the same letter are significantly different.

CA_E_ did not show any clear CO_2_-related trend in G1-1. In G1-2, CO_2_ clearly stimulated cell expansion in mature leaves with a CA_E_ of 939.47 μm^2^ at 150 ppmv, 1036.75 cm^2^ at 450 ppmv, and 1233.97 cm^2^ at 800 ppmv. The strong relationship between LA and CA_E_, as well as the strong relation to CO_2_, is visualised in Fig 1. Accordingly, the enhanced cell expansion is indicated with lower D_E_ across the CO_2_ treatments (Fig 2A).

Stomatal geometry is not significantly related to CO_2_ concentration in G1-1 or G1-2. Mean L_P_ ranges from 18.9 μm to 19.6 μm while mean L_S_ ranges between 28.0 μm and 29.6 μm and mean L_GC_ of around 9.3 μm in all treatments except at 150 ppmv where L_GC_ is slightly larger at 10.5 μm.

D_S_ was highest at low CO_2_ and decreased successively with CO_2_ increase in each of the experimental treatments. The decrease was significant in the step between 162 mm^-2^ at 150 ppmv to 136 mm^-2^ at 800 ppmv (Fig 2B). SI, calculated from D_E_ and D_S_, did not have a pronounced response to experimental CO_2_ conditions in G1-2 (Fig 2C). The absence of a CO_2_ response of SI in this case was a result of the parallel lowering of D_E_ and D_S_ across the experimental CO_2_ treatments. Mean g_smax_ did not vary significantly between the CO_2_ treatments, however a weak lowering of mean g_smax_ between 150 ppmv to both 450 and 800 ppmv was observed (Fig 2D).

### Leaf response to CO_2_ in G2

The leaf morphological characteristics and stomatal parameters in the subsequent (2014) growth season, G2, with the same individual plants from the initial experiment, were examined. The repeated exposure during the growing season to experimental CO_2_ conditions allowed for the evaluation of the response of leaves to experimental CO_2_ from two consecutive growing seasons.

In G2, the smallest leaves were produced at 150 ppmv with a mean LA of 0.58 cm^2^ compared to 1.19 cm^2^ at 450 ppmv, 1.22 cm^2^ at 800 ppmv, and 1.04 cm^2^ in the 399 ppmv control treatment (Fig 1A). Mean LA was not significantly different between the ambient experimental CO_2_ chamber (450 ppmv) and the control greenhouse (399 ppmv). CA_E_ was lowest at 150 ppmv and significantly higher at both 450 and 800 ppmv, but not significantly different in the step between 450 and 800 ppmv. D_E_ is significantly lower at 150 ppmv, with a mean of 852.8 mm^-2^, than in all other treatments. D_E_ did not significantly differ in the step between 450 and 800 ppmv, nor between 450 ppmv and the 399 ppmv control greenhouse treatment (Fig 2A).

In G2, mean D_S_ was higher in the 150 ppmv chamber with values of 152 mm^-2^ compared to 450 and 800 ppmv where means of 122 mm^-2^ and 123 mm^-2^ were observed, although this difference is not significant (Fig 2B). Plants in the 399 ppmv control greenhouse treatment, however, produced leaves with a significantly higher D_S_ of 133 mm^-2^ compared to plants in the 450 ppmv experimental CO_2_ chamber.

SI responded significantly to CO_2_ at 150 ppmv compared to the 450 and 800 ppmv CO_2_. SI at 150 ppmv was 15.2%, while at 450 ppmv it was 10.5% and at 800 ppmv, 10.3%. No significant difference in SI between 450 ppmv and the 399 ppmv greenhouse control, where SI was 11.16%, were observed (Fig 2C).

The g_smax_ of 1.04 mol m^-2^ s^-1^ at 150 ppmv was significantly higher than g_smax_ of 0.71 mol m^-2^ s^-1^ at 450 ppmv and 0.66 mol m^-2^ s^-1^ at 800 ppmv. In the 399 ppmv greenhouse control treatment, g_smax_ was significantly higher than in the 450 ppmv experimental CO_2_ chamber, measuring 0.90 mol m^-2^ s^-1^ (Fig 2D).

Stomatal geometry adjusted to experimental CO_2_ conditions in G2. L_P_ was significantly larger at 150 ppmv, measuring 19.9 μm compared to 17.4 μm at 450 ppmv and 17.3 μm at 800 ppmv. No significant difference was observed between the 450 ppmv and control greenhouse treatment where mean L_P_ was 17.5 μm. L_S_ was highest at 150 ppmv, measuring 32.4 μm, which was significantly larger than L_S_ of 28.9 μm at 450 ppmv. L_S_ was slightly, but insignificantly, larger at 800 ppmv than at 450 ppmv, measuring 30.7 μm. L_S_ was not significantly different between 450 ppmv and the greenhouse control at 27.3 μm. L_GC_ did not vary significantly between experimental CO_2_ treatments. L_GC_ was significantly smaller in the 399 ppmv control greenhouse treatment, measuring 7.0 μm, compared to 450 ppmv where mean L_GC_ measured 9.3 μm.

## Discussion

Changes in leaf morphology and structural stomatal parameters in response to experimental CO_2_ exposure were observed between G1-1 and G1-2 and between G1 and G2. The adjustment of different leaf parameters occured on a range of time scales; these are discussed in the following sections.

### Leaf ontogeny in *B*. *nana* in G1

The G1 data was divided into a first and second leaf flush, G1-1 and G1-2, respectively (Tables 2 and 3). The second leaf flush, G1-2, was initiated and formed entirely in the growth chambers under constant environmental and CO_2_ conditions, and it was thus possible to evaluate the response of leaf morphology and stomatal properties solely related to experimental CO_2_ exposure. With constant growth conditions we excluded the potential influence of, e.g., higher temperature or changing light conditions during the growth season which potentially alter leaf ontogeny and stomatal expression in naturally-grown lammas leaves [29].

### Leaf response in G1-1

Of the plastic and non-plastic leaf morphological parameters evaluated in this experiment, no conclusive or highly significant changes were observed in G1-1. LA was largest at 800 ppmv CO_2_ and L_P_ is marginally smaller at 150 ppmv. Other stomatal and epidermal cell frequency-related parameters were variable in the experimental CO_2_ chambers in G1-1, but did not reveal any significant structural trends related to CO_2_.

The absolute number of stomata expressed on the leaf surface is fixed before initiation of lateral leaf growth and is not sensitive to CO_2_ during leaf ontogeny [30]. Higher D_S_ at 150 ppmv in G1-1 compared to the other CO_2_ levels was likely a result of the intrinsic variability of stomatal expression rather than a true adjustment to experimental CO_2_ conditions, emphasising the required care when interpreting data from experiments of short duration with individual, pre-grown perennial plants.

### Leaf response in G1-2

At sampling day 73 significantly lower LA was associated with lower CA_E_ and higher D_E_ and D_S_. The combination of these parameters demonstrated the development of the next leaf generation, G1-2, which was formed *in situ* and is fully mature at 80–94 days, the sampling dates on which the data for leaf generation G1-2 were based.

From G1-2 on, a clearer response to CO_2_ in the plastic and non-plastic leaf and stomatal parameters was observed. LA increased linearly under successively higher CO_2_, forced by increasing lateral cell expansion which was expressed in the higher CA_E_ and lower D_E_. CO_2_ enrichment is associated with an increase in LA in a range of C3 species [31–34]

The extension of this trend to sub-ambient CO_2_ levels, however, is less well-documented in the literature but has been demonstrated for *Arabidopsis thaliana* grown under 100 ppmv and 380 ppmv where low CO_2_ structurally limited leaf expansion [35]. Along the experimental gradient in our study, D_S_ and D_E_ decreased in G1-2, although only the difference between 150 ppmv and 800 ppmv was significant. The trend in D_S_ was seemingly consistent with the expectation that increased CO_2_ concentration induced a lowering of stomatal frequency, however, in this case a large part must be attributed to higher D_E_ in the experimental treatments. Although Li et al. [35] propose the structurally higher D_S_ in *A*. *thaliana* was related to their 100 ppmv CO_2_ treatment, this signal was likely also—at least partially—related to the extremely restricted leaf expansion at 100 ppmv CO_2_ rather than a true response to the CO_2_ signal.

No significant response of SI or g_smax_ to CO_2_ was observed in G1-2, however a small but insignificant reduction in g_smax_ over the experimental CO_2_ gradient was observed.

### Leaf response in G2

The consecutive 2014 growth season, G2, allowed us to evaluate the multi-seasonal response of stomatal properties to long-term controlled-CO_2_ exposure, which may not be evident in a single-season CO_2_ experiment. The adjustment of plastic LA as a CO_2_ response also occurred in G2 with significantly smaller leaves produced at 150 ppmv than at 450 ppmv and 800 ppmv. As in G1, low LA at 150 ppmv was associated with low CA_E_ and higher LA with higher CA_E_ at 450 ppmv and 800 ppmv (Fig 1B) showing that lateral epidermal cell expansion was strongly regulated by CO_2_ availability as in G1.

In contrast with G1, G2 revealed pronounced responses of non-plastic structural stomatal parameters D_S_, SI, and g_smax_ to CO_2_ along the CO_2_ gradient between 150, 399, and 450 ppmv CO_2_. The additional enrichment step from 450 to 800 ppmv, however, demonstrated response levelling-off with only minor additional changes compared to the step from low to ambient CO_2_ conditions in all relevant parameters.

In studies of European tree birches, *Betula pendula* and *Betula pubescens*, analysis of SI in leaves grown over the industrial CO_2_ increase from 290 to 360 ppmv showed that these species reach their upper CO_2_ response limit at around 400 to 430ppmv [36]. This hypothesis is based on the observed successive slowdown in SI decrease from ~340ppmv onwards. A comparable pattern was observed in herbarium and modern leaf specimens collected over the period from 1919 to 2002, where the SI response levelled off between ~ 350 to 380 ppmv [11]. Moreover, available data from single-season elevated CO_2_ experiments and variable nitrogen treatments did not show any significant SI decline over the CO_2_ doubling from 350 ppmv to 700 ppmv in *B*. *pendula* [37]. Our data fully support the assertion that much of the stomatal response of *B*. *nana* occurs between 150 and 399 ppmv, with some further adjustment between 399 and 450 ppmv and no additional SI decrease between 450 and 800 ppmv. These observations of response patterns in SI hold for D_S_ in all studies. After pronounced initial decline over the experimental gradient from sub-ambient to ambient CO_2_, D_S_ and SI levelled off. Stomatal frequency adaptation is species-specific and should be evaluated for individual species; in the case of European tree and shrub birches, however, the CO_2_ ceiling of the SI at around 400 ppmv is a common feature which occurs independently in naturally-grown as well as in experimental leaf material.

From D_S_ and stomatal geometry, structural maximum stomatal conductance, g_smax_, was derived; this is an important hydrological parameter regulating the water exchange between plant and atmosphere [13,14,28]. C3 plants reduce their transpirational water loss in response to increasing atmospheric CO_2_ concentrations, which may affect global climate by reduced cloud formation and precipitation, thus exerting a physiological feedback on climate and hydrology [38–40].

In *B*. *nana*, g_smax_ showed a strong adjustment to CO_2_ in G2 compared to G1 with over 30% reduction of g_smax_ in G2 from 150 ppmv to 450 ppmv. The additional CO_2_ increase to 800 ppmv induced only a ~-6% further reduction in g_smax_. Comparing our results to stomatal conductance to water vapour (g_wmax_) data deduced from historical *B*. *nana* leaf material [12], we calculated structurally lower absolute values which may, however, result from different input parameters used in the g_smax_ calculation for each dataset. In Florida species collected over the past 150 years from herbarium and naturally-grown specimens, a reduction in g_smax_ of ~-33% per 100 ppmv for angiosperms and ~-37% per 100 ppmv for conifers was observed [14]. In the Florida study, long-term species adaptation within the limits of phenotypic plasticity was a result of the plants primarily adjusting D_S_, and to some extent stomatal dimensions, as a response to the anthropogenic CO_2_ increase [13]. Like these species, experimentally-grown *B*. *nana* from our study adjusted D_S_ and, to some extent, stomatal architecture within its phenotypic plasticity in response to changing CO_2_ conditions, in order to optimise CO_2_ uptake and reduce transpirational water loss.

One possible way to compare the various datasets is by evaluating the relative response rate of g_smax_ to CO_2_. In our *B*. *nana* study this is -0.11% ppmv^-1^ from 150 to 450 ppmv and -0.06% ppmv^-1^ between 150 and 800 ppmv. This decline in g_smax_ corresponds with observed rates of change of -0.16% ppmv^-1^ in naturally grown *B*. *nana* over the industrial CO_2_ increase of 290 to 380 ppmv [12]. In growth experiments with *Nothofagus fusca*, response rates between -0.21 and -0.31% ppmv^-1^ were observed over a 260 to 370 ppmv CO_2_ gradient [Hincke et al., *unpub*.]. Relatively few studies have been conducted where g_smax_ is inferred from leaf cuticle analysis of material from growth experiments, herbaria, and well-dated palaeo-cores. The data from G2 is within the range of published response rates, between -0.13 and -0.47% ppmv^-1^ (see S1 Table for a summary of reported response rates of g_smax_ in a range of species).

### Stomatal adjustment over successive growth seasons

In response to changing atmospheric CO_2_, plants adjust stomata and leaf diffusive conductance to optimise CO_2_ uptake and limit water loss [41]. The adjustment, however, does not occur instantaneously and takes place over multiple seasons, although some plastic leaf parameters, such as LA, may adjust within one season of exposure to experimental CO_2_ conditions. Our data suggest that the majority of stomatal response occurred between 150 and 450 ppmv, but not before a new leaf generation formed completely under experimental conditions, indicating that either the upper phenotypic response limits for this species were reached around 400–450 ppmv, or that no further adjustment was necessary to limit water loss as a response to CO_2_ enrichment past this level. Data from subsequent experimental growth seasons is required to evaluate whether the response limit for *B*. *nana* was achieved in G2 in this experiment, or if further adjustments of SI, g_smax_, and structural stomatal parameters as a response to CO_2_ would occur in G3 onwards.

The data from consecutive growth seasons G1 and G2 in *B*. *nana* strongly emphasise the requirement for multi-generational growth experiments with the same plant individuals to observe the full plant response to CO_2_ conditions. The single-season nature of previous experimental work may be an insufficient length of time to evaluate the true response of structural stomatal parameters to CO_2_ change.

Lammas leaves provide a method to test the CO_2_ acclimation response within one season of growth as they are initiated during the growth season under experimental CO_2_ conditions. Some adjustment of leaf parameters in the lammas generation (G1-2) was observed in *B*. *nana* with adjustment of D_S_, D_E_, and CA_E_, however, key structural parameters SI and g_smax_ did not respond clearly to CO_2_ in a single experimental season. In *A*. *thaliana* newly formed leaves produced significantly lower D_S_ and SI when mature leaves were exposed in cuvettes to elevated CO_2_ conditions [24], a similar leaf generation response to the effect observed from G1 to G2 in *B*. *nana*.

While free-air carbon enrichment (FACE) experiments found some reduction in stomatal properties D_S_ and g_smax_ under elevated CO_2_ conditions in some studies, the observed reduction in g_smax_ was attributed to instantaneous adaptation and not a long-term CO_2_ effect [17,18,42,43]. Studies conducted evaluating plant response to ambient and elevated CO_2_ may not capture the full range of structural stomatal response to CO_2_ as the majority of reduction in D_S_, SI, and g_smax_ occurs below the species response limits of ~400–450 ppmv.

Earlier experiments with *B*. *pendula* found no clear response in SI, measured over one leaf generation, to elevated CO_2_ exposure [37]. The lack of SI response in this case was due to the single-season nature of the experiment. Seedlings were germinated, and the leaves initiated, under global ambient CO_2_ for 40 days prior to exposure to experimental CO_2_ levels [44]. SI adjustment occurred between 150 and 399 ppmv in our data, and as the *B*. *pendula* study examined ambient to high CO_2_ conditions, it is possible that no further adjustment of SI to CO_2_ occurred past the species response limits of c. 400 ppmv, and thus was not be captured in the *B*. *pendula* study. Single-season experiments such as the *B*. *pendula* study potentially do not capture the full CO_2_ range under which stomatal acclimation occurs, rather, they likely capture changes resulting from plants adjusting within their phenotypic plasticity which does not reflect a true, long-term adjustment. Our results demonstrate that true leaf-level adjustment of stomatal properties in response to altered CO_2_ levels takes place over at least two successive growth seasons.

### Implications of the long-term experimental approach for D_S_- and SI-based CO_2_ reconstructions

In G2, both D_S_ and SI were responsive to CO_2_ across the extensive range of glacial CO_2_ lows to potential future CO_2_ highs. The absolute values of D_S_ observed in the experimental data were lower than D_S_ from historical *B*. *nana* data, which have values between 300 mm^-2^ for pre-industrial CO_2_ levels and approaches 100 mm^-2^ around 370 ppmv CO_2_ [11]. SI ranged from 15% in experimental *B*. *nana* grown in the 150 ppmv chamber to 11.2% at 399 ppmv, to 10.6% at 450 ppmv and 10.3% at 800 ppmv. Historical SI values of 12% at pre-industrial CO_2_ concentrations of around 290 ppmv to 9–5.5% at around 370 ppmv CO_2_ levels are recorded in the literature [11]. The 15% SI from experimental *B*. *nana* grown at 150 ppmv would logically extend the dataset to glacial CO_2_ conditions, however SI at ambient to high CO_2_ range are higher than expected compared to the historical dataset. Differences in measured parameters between historical and experimentally-grown *B*. *nana* may be due to habitat variability between the tested populations or the difference in growth conditions between historical specimens and the growth chamber experiments. In our experiments, the systematic down-regulation of parameter SI was directly related to CO_2_ due to the consistent conditions in the experimental set-ups. In a study with *B*. *pendula*, SI linearly increased in 12°C, 20°C, and 30°C treatments [37]. In our study, SI at 12°C (11%) was still significantly higher than 8.4% SI observed under natural growth conditions at 10°C mean annual temperature in the southern Netherlands [37]. While some offset between experimentally-grown *B*. *nana* and naturally-grown sub-arctic plants and growth chamber plants is expected due to higher temperature conditions in the growth chambers compared to natural sub-Arctic conditions, these systematic offsets in absolute values were related to specific experimental conditions and not the response of stomatal parameters to CO_2_ conditions.

Work with herbarium and down-core sub-fossil leaves and leaf fragments provide the potential for seasonally-resolved stomatal data calibrated against instrumental measurements. Data collected year-to-year automatically cover multiple generations and growth seasons. Where material from the same individual plant or genetic plant population was sampled the adjustment of stomatal parameters can be traced over known atmospheric CO_2_ ranges. However, these ranges are limited to the CO_2_ rise between pre-industrial CO_2_ of approximately 290 ppmv and present values of 400 ppmv, and exclude glacial CO_2_ conditions and potential future CO_2_ rise. Incorporating growth chamber data into historical herbarium and well-dated palaeo material datasets can provide a better understanding of plant response to varying atmospheric CO_2_ levels through the Cenozoic CO_2_ range.

### Comparison of greenhouse and growth chamber conditions

The greenhouses which housed the control (399 ppmv) plants during this study were exposed to open air and natural light conditions through a semi-shaded roof. In the experimental chambers, artificial light conditions and humidity were more strictly controlled. The temperature was controlled in both set-ups (~20°C in the greenhouses compared to 21°C/18°C day/night in growth chambers) so this factor was unlikely to cause any offset in measured parameters. While there was a small offset in measured stomatal parameters between the greenhouse control and ambient experimental growth chamber, the control values of morphology and stomatal features from the greenhouse-grown plants did not significantly differ from the plants grown in the ambient CO_2_ growth chamber. This confirms that, despite differences in the environmental conditions in both set-ups, for example, exposure to the natural diurnal light cycle in the greenhouses rather than artificial light in the growth chambers, it may still be possible to integrate data generated from long-term experimentally-grown plants with datasets for validation from herbaria or sub-fossil leaf deposits from peat sequences.

## Conclusions

Results from our growth experiments with *B*. *nana* showed that while a short-term response to CO_2_ change over one growing season was evident in leaf morphological and stomatal parameters including LA, D_S_, and D_E_, the full effect of exposure to experimental CO_2_ conditions, especially on non-plastic stomatal properties g_smax_ and SI, was not captured in G1 as these parameters did not fully adjust to CO_2_ until G2. The leaf-level signal may thus be skewed if leaf sampling occurs during the early stages of ontogeny, potentially concealing the true CO_2_ response in these plants. Our results validate the seasonal-scale response of leaf morphological parameter LA and stomatal parameters D_S_ and D_E_ to CO_2_ in *B*. *nana*, as postulated by Lake et al. [24].

Further, we confirm that the intensified adjustment or acclimation response of stomatal parameters occurs over several growing seasons under altered CO_2_. Our data improves the understanding of seasonal response of stomatal parameters in a woody C3 plant by demonstrating that the stomatal response to atmospheric CO_2_ in *B*. *nana* extends over a minimum of two consecutive growth seasons within one population of plants. The documented response provides a likely explanation to the—so far—partially contradictory results between historical and palaeobotanical records and experimental data. It is not possible within the context of this experiment to predict whether the observed response is the full extent of the adjustment of *B*. *nana* to experimental CO_2_ conditions, as further adjustments to stomatal properties may continue in subsequent growing seasons. Our findings very strongly support the necessity for multi-annual experiments to fully quantify the extent of stomatal acclimation to the range of Cenozoic CO_2_ concentrations in perennial C3 species as short-term growth experiment data may not reflect the full morphological and stomatal response to experimental CO_2_.

## Supporting Information

S1 Fig Specific leaf area (SLA, cm^-2^ g^-1^) for G1-2 and G2 in each CO_2_ treatment. The error is standard error of the mean. No clear pattern of response of this parameters in G1-2 was observed. In G2, SLA was lower at higher CO_2_ levels, with a levelling-off of the response between 450 ppmv and 800 ppmv.
(TIF)

S1 Table The g_smax_ response rates to a CO_2_ gradient observed in a range of species. The response rates of g_smax_ of species studied in experimental set-ups and from herbarium records was calculated as % ppmv^-1^. The response rate of g_smax_ is negative with increasing CO_2_ in all cases.
(PDF)
