# Supplementary figures and images for: Multi-Year Leaf-Level Response to Sub-Ambient and Elevated Experimental CO2 in Betula nana
Source: PLoS One. 2016 Jun 10;11(6):e0157400. doi: 10.1371/journal.pone.0157400 (PMC4902311; doi:10.1371/journal.pone.0157400)

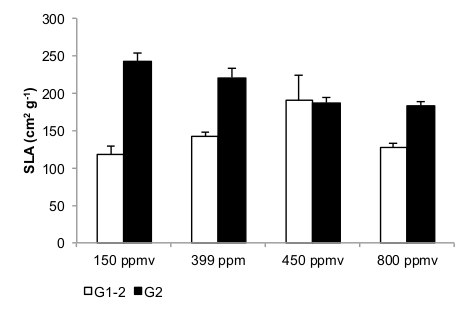

Supplement: S1 Fig — The error is standard error of the mean. No clear pattern of response of this parameters in G1-2 was observed. In G2, SLA was lower at higher CO2 levels, with a levelling-off of the response between 450 ppmv and 800 ppmv. (TIF) [file pone.0157400.s001.tif]
