# Supplementary material for: Multi-Year Leaf-Level Response to Sub-Ambient and Elevated Experimental CO2 in Betula nana
Source: PLoS One. 2016 Jun 10;11(6):e0157400. doi: 10.1371/journal.pone.0157400 (PMC4902311; doi:10.1371/journal.pone.0157400)
Supplement: S1 Table — The response rates of gsmax of species studied in experimental set-ups and from herbarium records was calculated as % ppmv-1. The response rate of gsmax is negative with increasing CO2 in all cases. (PDF) [file pone.0157400.s002.pdf]

**Species**	**Study**	**CO_2 range**	**% ppmv-1**
Betula nana	G2, this study	150—800	-0.06
		150—450	-0.11
	Gagen et al. 2011	290—380	-0.16
Nothofagus fusca	Hincke, *submitted*	260—370	-0.21 — -0.31
		260—650	-0.05 — -0.12
Sub-tropical (range)	Lammertsma et al. 2011	290—390	-0.17 — -0.42
Abutilon theophrasti	Bunce 2007	100—380	-0.18
Glycine max	Bunce 2007	100—380	-0.15
Gossypium hirsutum	Bunce 2007	100—380	-0.13
Xanthium strumarium	Bunce 2007	100—380	-0.15
Helianthus annuus	Rico et al. 2013	290—390	-0.22
		390—480	-0.17
		290—480	-0.18
Phaseolus vulgaris	Cowling & Sage 1998	200—380	-0.15 — -0.27
		200—380	-0.15
Solanum dimidiatum	Maherali et al. 2002	200—550	-0.23
Bromus japonicus	Maherali et al. 2002	215—550	-0.15
